# *N*-[2-(4-Methyl­benzo­yl)eth­yl]propan-2-aminium chloride

**DOI:** 10.1107/S1600536812035271

**Published:** 2012-08-15

**Authors:** Abdullah Aydın, Mehmet Akkurt, Halise Inci Gul, Ebru Mete, Ertan Sahin

**Affiliations:** a Department of Science Education, Faculty of Education, Kastamonu University, 37200 Kastamonu, Turkey; b Department of Physics, Faculty of Sciences, Erciyes University, 38039 Kayseri, Turkey; c Department of Pharmaceutical Chemistry, Faculty of Pharmacy, Atatürk University, 25240 Erzurum, Turkey; d Department of Chemistry, Faculty of Sciences, Atatürk University, 25240 Erzurum, Turkey

## Abstract

In the title compound, C_13_H_20_NO^+^·Cl^−^, the protonated amino N atom is hydrogen bonded to the chloride anion. N—H⋯Cl hydrogen bonds link the anions and cations into dimers, which are connected by C—H⋯O hydrogen bonds, forming supra­molecular chains extending along [100].

## Related literature

For the details of the pharmacological effects of Mannich bases and for their synthesis, see: Dimmock & Kumar (1997 ▶); Gul *et al.* (2004 ▶; 2005*a* ▶,*b* ▶; 2009 ▶); Gul (2005 ▶); Mete *et al.* (2011*a* ▶,*b* ▶); Kucukoglu *et al.* (2011 ▶); Canturk *et al.* (2008 ▶); Chen *et al.* (1991 ▶); Suleyman *et al.* (2007 ▶); Plastino *et al.* (1962 ▶, 1964 ▶). For bond-length data, see: Allen *et al.* (1987 ▶). For hydrogen-bond motifs, see: Bernstein *et al.* (1995 ▶); Etter (1990 ▶). For some related structures, see: Abonia *et al.* (2011 ▶); Tuzina *et al.* (2006 ▶).
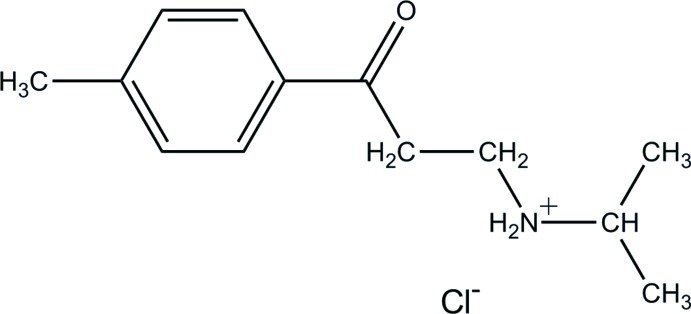


## Experimental

### 

#### Crystal data

C_13_H_20_NO^+^·Cl^−^
*M* *_r_* = 241.75
Monoclinic, 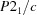

*a* = 7.786 (5) Å
*b* = 7.511 (5) Å
*c* = 23.365 (5) Å
β = 95.362 (5)°
*V* = 1360.4 (13) Å^3^
*Z* = 4
Mo *K*α radiation
μ = 0.26 mm^−1^
*T* = 294 K
0.17 × 0.11 × 0.10 mm

#### Data collection

Rigaku R-AXIS RAPID-S diffractometer
Absorption correction: multi-scan (Blessing, 1995 ▶) *T*_min_ = 0.966, *T*_max_ = 0.974
26900 measured reflections
2800 independent reflections
2007 reflections with *I* > 2σ(*I*)
*R*_int_ = 0.066

#### Refinement

*R*[*F* ^2^ > 2σ(*F* ^2^)] = 0.049
*wR*(*F* ^2^) = 0.151
*S* = 1.06
2800 reflections
149 parameters
H-atom parameters constrained
Δρ_max_ = 0.21 e Å^−3^
Δρ_min_ = −0.18 e Å^−3^

### 

Data collection: *CrystalClear* (Rigaku/MSC, 2005 ▶); cell refinement: *CrystalClear*; data reduction: *CrystalClear* (Rigaku/MSC, 2005 ▶); program(s) used to solve structure: *SHELXS97* (Sheldrick, 2008 ▶); program(s) used to refine structure: *SHELXL97* (Sheldrick, 2008 ▶); molecular graphics: *ORTEP-3 for Windows* (Farrugia, 1997 ▶) and *PLATON* (Spek, 2009 ▶); software used to prepare material for publication: *WinGX* (Farrugia, 1999 ▶).

## Supplementary Material

Crystal structure: contains datablock(s) global, I. DOI: 10.1107/S1600536812035271/qm2078sup1.cif

Structure factors: contains datablock(s) I. DOI: 10.1107/S1600536812035271/qm2078Isup2.hkl

Supplementary material file. DOI: 10.1107/S1600536812035271/qm2078Isup3.cml

Additional supplementary materials:  crystallographic information; 3D view; checkCIF report

## Figures and Tables

**Table 1:** Hydrogen-bond geometry (Å, °)

*D*—H⋯*A*	*D*—H	H⋯*A*	*D*⋯*A*	*D*—H⋯*A*
N1—H14*A*⋯Cl1	0.90	2.26	3.148 (3)	172
N1—H14*B*⋯Cl1^i^	0.90	2.25	3.145 (3)	173
C1—H1⋯O1^ii^	0.93	2.53	3.340 (4)	146

Symmetry codes: (i) 
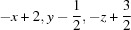
; (ii) 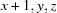.

**Fig. 1.**
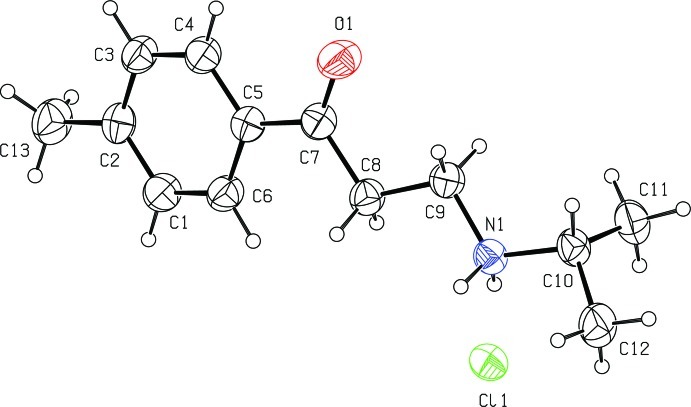
The title molecule with the atom numbering scheme. Displacement ellipsoids fornon-H atoms are drawn at the 30% probability level.

**Fig. 2.**
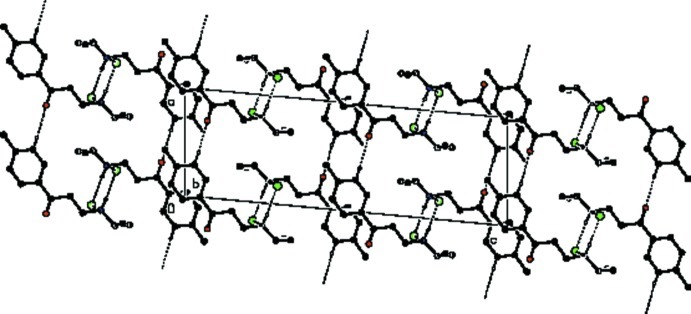
The packing and hydrogen bonding of the title compound viewed down the baxis. H atoms not involved in hydrogen bondings are omitted for clarity.

## supplementary crystallographic information

### Comment

Mannich bases are generally formed by the reaction between formaldehyde, a secondary amine and a compound containing reactive hydrogen atoms. On occasion, aldehydes other than formaldehyde may be employed and the secondary amine may be replaced by ammonia and primary amines. This process is known as the Mannich reaction (Dimmock & Kumar, 1997).

Mannich bases display varied biological activities such as antimicrobial (Gul *et al.*, 2005; Mete *et al.*, 2011*a*), cytotoxic (Gul *et al.*, 2005; Mete *et al.*, 2011*b*; Kucukoglu *et al.*, 2011; Canturk *et al.*, 2008), anticancer (Dimmock & Kumar, 1997; Chen *et al.*, 1991; Gul, 2005), antiinflammatory (Suleyman *et al.*, 2007; Gul *et al.*, 2009), anticonvulsant (Gul *et al.*, 2004) and DNA topoisomeraseI inhibiting properties (Canturk *et al.*, 2008).

In the title compound (I), (Fig. 1), bond lengths and bond angles are within the range of expected values for this type of compound (Allen *et al.*, 1987; Abonia *et al.*, 2011; Tuzina *et al.*, 2006). The protonated N1 atom forms a hydrogen bond to Cl1 (Table 1).

Intra- and intermolecular N—H···Cl hydrogen-bonding interactions between the free chloride anion and the organic cation link the molecules into hydrogen-bond dimers, forming a *R*^2^_2_(6) motif (Bernstein *et al.*, 1995; Etter, 1990). The dimers are connected by C—H···O hydrogen bonds into chains extended along the *a* axis (Table 1, Fig. 2).

### Experimental

A mixture of the appropriate ketone (50 mmol), paraformaldehyde (50 mmol), and isopropylamine hydrochloride (27 mmol) was heated in an oil bath at 403 K. The reaction vessel was then removed from the oil bath and when the temperature of the mixture dropped to 338 K, ethyl acetate (40–80 ml) was added. The mixture was stirred at room temperature for 24 h and the resultant precipitate was then collected and were recrystallized from ether/methanol. The melting point and yield of this compound was: 443–444 K (lit. Plastino *et al.*, 1964 m.p. 444–445 K), 58% (Mete *et al.*, 2011*b*).

^1^H-NMR δ 1.50 (d, J = 6.6 Hz, 6H, CH(CH_3_)_2_), 2.34 (s, 3H, ArCH_3_), 3.36–3.46 (m, 3H, CH(CH_3_)_2_ and 2 *x* H-2), 3.74 (t, J = 7.3 Hz, 2H, 2 *x* H-3), 7.14 (d, J = 8.1 Hz, 2H, H-3'/5'), 7.79 (d, J = 8.1 Hz, 2H, H-2'/6'), 9.54 (brs, 2H, NH_2_^+^); ^13^C-NMR δ 19.4 (CH(CH_3_)_2_), 21.9, 35.1, 40.5, 51.2, 128.5, 129.6, 133.6, 144.9, 196.6; MS (EI) m/*z* (%): 190.1 (M–CH_3_)^+^, 205.3 (*M*^+^). IR (KBr, cm^-1^): 2461 (NH_2_^+^), 1679 (CO). Calcd. for C_13_H_20_ClNO (241.76): C, 64.59: H, 8.34; N, 5.79. Found: C, 64.39; H, 8.45; N, 5.53.

### Refinement

H atoms were positioned geometrically, with N—H = 0.90 Å, C—H = 0.93(aromatic), 0.97(methylene) and 0.98 Å (methine), and refined as riding with *U*_iso_(H) = 1.5*U*_eq_(O) for methyl H and 1.2*U*_eq_(C) for the others.

### Crystal data

**Table:**

C_13_H_20_NO^+^·Cl^−^	*F*(000) = 520
*M**_r_* = 241.75	*D*_x_ = 1.180 Mg m^−^^3^
Monoclinic, *P*2_1_/*c*	Mo *K*α radiation, λ = 0.71073 Å
Hall symbol: -P 2ybc	Cell parameters from 4594 reflections
*a* = 7.786 (5) Å	θ = 2.6–26.5°
*b* = 7.511 (5) Å	µ = 0.26 mm^−^^1^
*c* = 23.365 (5) Å	*T* = 294 K
β = 95.362 (5)°	Block, white
*V* = 1360.4 (13) Å^3^	0.17 × 0.11 × 0.10 mm
*Z* = 4	

### Data collection

**Table:**

Rigaku R-AXIS RAPID-S diffractometer	2800 independent reflections
Radiation source: Sealed Tube	2007 reflections with *I* > 2σ(*I*)
Graphite Monochromator monochromator	*R*_int_ = 0.066
Detector resolution: 10.0000 pixels mm^-1^	θ_max_ = 26.4°, θ_min_ = 2.6°
dtprofit.ref scans	*h* = −9→9
Absorption correction: multi-scan (Blessing, 1995)	*k* = −8→9
*T*_min_ = 0.966, *T*_max_ = 0.974	*l* = −29→29
26900 measured reflections	

### Refinement

**Table:**

Refinement on *F*^2^	Secondary atom site location: difference Fourier map
Least-squares matrix: full	Hydrogen site location: inferred from neighbouring sites
*R*[*F*^2^ > 2σ(*F*^2^)] = 0.049	H-atom parameters constrained
*wR*(*F*^2^) = 0.151	*w* = 1/[σ^2^(*F*_o_^2^) + (0.0681*P*)^2^ + 0.2172*P*] where *P* = (*F*_o_^2^ + 2*F*_c_^2^)/3
*S* = 1.06	(Δ/σ)_max_ < 0.001
2800 reflections	Δρ_max_ = 0.21 e Å^−^^3^
149 parameters	Δρ_min_ = −0.18 e Å^−^^3^
0 restraints	Extinction correction: *SHELXL97* (Sheldrick, 2008), FC^*^=KFC[1+0.001XFC^2^Λ^3^/SIN(2Θ)]^-1/4^
Primary atom site location: structure-invariant direct methods	Extinction coefficient: 0.010 (3)

### Special details

**Table:**

Geometry. Bond distances, angles *etc*. have been calculated using the rounded fractional coordinates. All su's are estimated from the variances of the (full) variance-covariance matrix. The cell e.s.d.'s are taken into account in the estimation of distances, angles and torsion angles
Refinement. Refinement on *F*^2^ for ALL reflections except those flagged by the user for potential systematic errors. Weighted *R*-factors *wR* and all goodnesses of fit *S* are based on *F*^2^, conventional *R*-factors *R* are based on *F*, with *F* set to zero for negative *F*^2^. The observed criterion of *F*^2^ > σ(*F*^2^) is used only for calculating -*R*-factor-obs *etc*. and is not relevant to the choice of reflections for refinement. *R*-factors based on *F*^2^ are statistically about twice as large as those based on *F*, and *R*-factors based on ALL data will be even larger.

### Fractional atomic coordinates and isotropic or equivalent isotropic displacement parameters (Å^2^)

**Table:**

	*x*	*y*	*z*	*U*_iso_*/*U*_eq_	
O1	0.7123 (2)	0.2422 (3)	0.57107 (8)	0.0903 (8)	
N1	0.7916 (2)	0.0622 (2)	0.74056 (7)	0.0555 (6)	
C1	1.2949 (3)	0.1740 (3)	0.53020 (10)	0.0722 (9)	
C2	1.2627 (3)	0.2488 (3)	0.47633 (10)	0.0653 (8)	
C3	1.0947 (3)	0.2961 (3)	0.45820 (9)	0.0680 (9)	
C4	0.9644 (3)	0.2737 (3)	0.49337 (9)	0.0659 (8)	
C5	0.9974 (3)	0.2024 (3)	0.54815 (9)	0.0563 (7)	
C6	1.1652 (3)	0.1501 (3)	0.56544 (10)	0.0658 (8)	
C7	0.8564 (3)	0.1895 (3)	0.58624 (9)	0.0616 (8)	
C8	0.8956 (3)	0.1133 (3)	0.64590 (9)	0.0623 (8)	
C9	0.7524 (3)	0.1489 (3)	0.68361 (9)	0.0606 (8)	
C10	0.6625 (3)	0.0981 (3)	0.78362 (9)	0.0611 (8)	
C11	0.6888 (4)	0.2830 (4)	0.80850 (12)	0.0807 (10)	
C12	0.6811 (4)	−0.0436 (4)	0.82927 (12)	0.0864 (10)	
C13	1.4074 (4)	0.2830 (5)	0.43891 (12)	0.0913 (11)	
Cl1	1.17234 (7)	0.15576 (7)	0.78840 (2)	0.0665 (2)	
H1	1.40660	0.13910	0.54290	0.0870*	
H3	1.06950	0.34370	0.42160	0.0820*	
H4	0.85250	0.30690	0.48030	0.0790*	
H6	1.19010	0.09810	0.60140	0.0790*	
H8A	0.91290	−0.01420	0.64310	0.0750*	
H8B	1.00180	0.16520	0.66350	0.0750*	
H9A	0.73980	0.27620	0.68870	0.0730*	
H9B	0.64450	0.10290	0.66530	0.0730*	
H10	0.54610	0.09070	0.76380	0.0730*	
H11A	0.59650	0.31080	0.83150	0.1210*	
H11B	0.68980	0.36790	0.77780	0.1210*	
H11C	0.79670	0.28780	0.83190	0.1210*	
H12A	0.66880	−0.15880	0.81160	0.1290*	
H12B	0.59360	−0.02790	0.85520	0.1290*	
H12C	0.79290	−0.03440	0.85020	0.1290*	
H13A	1.43820	0.40670	0.44090	0.1370*	
H13B	1.37010	0.25180	0.39990	0.1370*	
H13C	1.50570	0.21210	0.45220	0.1370*	
H14A	0.89610	0.09920	0.75560	0.0670*	
H14B	0.79790	−0.05620	0.73520	0.0670*	

### Atomic displacement parameters (Å^2^)

**Table:**

	*U*^11^	*U*^22^	*U*^33^	*U*^12^	*U*^13^	*U*^23^
O1	0.0562 (10)	0.1386 (17)	0.0763 (11)	0.0110 (11)	0.0077 (8)	0.0214 (11)
N1	0.0530 (10)	0.0541 (10)	0.0609 (10)	−0.0019 (8)	0.0135 (8)	0.0028 (8)
C1	0.0545 (13)	0.0940 (19)	0.0683 (15)	0.0016 (12)	0.0076 (11)	0.0037 (13)
C2	0.0651 (14)	0.0730 (15)	0.0596 (13)	−0.0076 (12)	0.0152 (10)	−0.0054 (11)
C3	0.0723 (16)	0.0783 (16)	0.0541 (13)	−0.0016 (12)	0.0094 (10)	0.0048 (11)
C4	0.0618 (14)	0.0764 (15)	0.0596 (13)	−0.0001 (12)	0.0057 (10)	0.0003 (11)
C5	0.0553 (12)	0.0579 (12)	0.0555 (12)	−0.0054 (10)	0.0048 (9)	−0.0023 (9)
C6	0.0625 (14)	0.0774 (16)	0.0577 (12)	0.0004 (11)	0.0066 (10)	0.0071 (11)
C7	0.0557 (13)	0.0684 (14)	0.0609 (13)	−0.0038 (11)	0.0059 (10)	−0.0008 (10)
C8	0.0574 (13)	0.0718 (14)	0.0588 (13)	−0.0014 (11)	0.0111 (10)	0.0039 (11)
C9	0.0584 (13)	0.0630 (14)	0.0614 (13)	0.0002 (10)	0.0103 (10)	0.0041 (10)
C10	0.0535 (12)	0.0672 (14)	0.0653 (13)	0.0004 (10)	0.0195 (10)	0.0012 (10)
C11	0.0901 (19)	0.0698 (16)	0.0871 (18)	0.0072 (13)	0.0350 (14)	−0.0041 (13)
C12	0.108 (2)	0.0767 (17)	0.0808 (17)	0.0039 (15)	0.0418 (15)	0.0138 (13)
C13	0.0797 (18)	0.120 (2)	0.0775 (18)	−0.0034 (17)	0.0253 (14)	0.0074 (16)
Cl1	0.0631 (4)	0.0604 (4)	0.0756 (4)	0.0010 (3)	0.0046 (3)	−0.0014 (3)

### Geometric parameters (Å, º)

**Table:**

O1—C7	1.211 (3)	C3—H3	0.9300
N1—C9	1.487 (3)	C4—H4	0.9300
N1—C10	1.511 (3)	C6—H6	0.9300
N1—H14B	0.9000	C8—H8A	0.9700
N1—H14A	0.9000	C8—H8B	0.9700
C1—C6	1.373 (3)	C9—H9A	0.9700
C1—C2	1.380 (3)	C9—H9B	0.9700
C2—C3	1.383 (3)	C10—H10	0.9800
C2—C13	1.511 (4)	C11—H11A	0.9600
C3—C4	1.374 (3)	C11—H11B	0.9600
C4—C5	1.389 (3)	C11—H11C	0.9600
C5—C7	1.480 (3)	C12—H12A	0.9600
C5—C6	1.388 (3)	C12—H12B	0.9600
C7—C8	1.511 (3)	C12—H12C	0.9600
C8—C9	1.508 (3)	C13—H13A	0.9600
C10—C12	1.504 (4)	C13—H13B	0.9600
C10—C11	1.512 (4)	C13—H13C	0.9600
C1—H1	0.9300		
			
C9—N1—C10	115.08 (16)	C7—C8—H8A	109.00
H14A—N1—H14B	107.00	C7—C8—H8B	109.00
C9—N1—H14B	109.00	C9—C8—H8A	109.00
C10—N1—H14A	109.00	C9—C8—H8B	109.00
C9—N1—H14A	108.00	H8A—C8—H8B	108.00
C10—N1—H14B	108.00	N1—C9—H9A	110.00
C2—C1—C6	121.3 (2)	N1—C9—H9B	110.00
C1—C2—C13	121.0 (2)	C8—C9—H9A	110.00
C1—C2—C3	118.0 (2)	C8—C9—H9B	110.00
C3—C2—C13	120.9 (2)	H9A—C9—H9B	108.00
C2—C3—C4	121.0 (2)	N1—C10—H10	109.00
C3—C4—C5	121.0 (2)	C11—C10—H10	109.00
C6—C5—C7	122.5 (2)	C12—C10—H10	109.00
C4—C5—C7	119.7 (2)	C10—C11—H11A	109.00
C4—C5—C6	117.7 (2)	C10—C11—H11B	109.00
C1—C6—C5	120.9 (2)	C10—C11—H11C	109.00
O1—C7—C5	121.5 (2)	H11A—C11—H11B	109.00
C5—C7—C8	118.8 (2)	H11A—C11—H11C	110.00
O1—C7—C8	119.7 (2)	H11B—C11—H11C	109.00
C7—C8—C9	112.03 (19)	C10—C12—H12A	109.00
N1—C9—C8	110.12 (18)	C10—C12—H12B	109.00
N1—C10—C12	108.65 (19)	C10—C12—H12C	109.00
C11—C10—C12	112.1 (2)	H12A—C12—H12B	110.00
N1—C10—C11	110.19 (19)	H12A—C12—H12C	109.00
C2—C1—H1	119.00	H12B—C12—H12C	109.00
C6—C1—H1	119.00	C2—C13—H13A	109.00
C2—C3—H3	119.00	C2—C13—H13B	109.00
C4—C3—H3	119.00	C2—C13—H13C	109.00
C3—C4—H4	119.00	H13A—C13—H13B	110.00
C5—C4—H4	120.00	H13A—C13—H13C	109.00
C1—C6—H6	120.00	H13B—C13—H13C	109.00
C5—C6—H6	120.00		
			
C9—N1—C10—C11	−76.7 (2)	C3—C4—C5—C7	176.5 (2)
C9—N1—C10—C12	160.08 (19)	C4—C5—C6—C1	2.2 (3)
C10—N1—C9—C8	176.31 (17)	C7—C5—C6—C1	−176.0 (2)
C6—C1—C2—C3	−1.3 (3)	C6—C5—C7—C8	−1.2 (3)
C6—C1—C2—C13	177.0 (2)	C4—C5—C7—O1	−1.1 (3)
C2—C1—C6—C5	−0.7 (4)	C4—C5—C7—C8	−179.4 (2)
C13—C2—C3—C4	−176.6 (2)	C6—C5—C7—O1	177.1 (2)
C1—C2—C3—C4	1.7 (3)	O1—C7—C8—C9	−11.9 (3)
C2—C3—C4—C5	−0.2 (3)	C5—C7—C8—C9	166.46 (19)
C3—C4—C5—C6	−1.8 (3)	C7—C8—C9—N1	176.54 (17)

### Hydrogen-bond geometry (Å, º)

**Table:**

*D*—H···*A*	*D*—H	H···*A*	*D*···*A*	*D*—H···*A*
N1—H14*A*···Cl1	0.90	2.26	3.148 (3)	172
N1—H14*B*···Cl1^i^	0.90	2.25	3.145 (3)	173
C1—H1···O1^ii^	0.93	2.53	3.340 (4)	146

Symmetry codes: (i) −*x*+2, *y*−1/2, −*z*+3/2; (ii) *x*+1, *y*, *z*.
